# Effectiveness of Chamomile in Reducing the Incidence and Severity of Nausea and Vomiting After Middle Ear Surgery: A Triple-Blind Randomized Study

**DOI:** 10.5812/aapm-153566

**Published:** 2025-01-07

**Authors:** Maryam Sarkhosh, Ehsan Rajabi Visroodi, Hamidreza Samaee, Motahareh Farhadi, Parisa Moradimajd

**Affiliations:** 1 Department of Anesthesia, Allied Medical School, Iran University of Medical Sciences, Tehran, Iran; 2 Student Research Committee, Faculty of Pharmacy, Mazandaran University of Medical Sciences, Sari, Iran; 3 Emam Sajjad Hospital, Iran University of Medical Sciences, Tehran, Iran

**Keywords:** Anesthesia, Chamomile, Middle Ear Surgery, Nausea, Postoperative Nausea and Vomiting, Vomiting

## Abstract

**Background:**

Nausea and vomiting are among the most common complications after surgery.

**Objectives:**

The aim of this study was to investigate the effectiveness of chamomile in reducing the incidence and severity of nausea and vomiting after middle ear surgery.

**Methods:**

A total of 110 patients who met the inclusion criteria were randomly assigned to either the chamomile or placebo group. Group A consumed chamomile drops (500 mg), while group B consumed cornstarch with 30 mL of water, one hour before surgery. The severity of nausea and frequency of vomiting were recorded at recovery (time of zero), 1, 2, 4, and 6 hours after surgery using the Rhodes Index. Data were analyzed using SPSS v.21 software, paired *t*-tests, and chi-square tests.

**Results:**

A total of 110 patients were included, with an average age of 36.14 ± 10.3 years (group A) and 34.28 ± 13.3 years (group B). There was no statistically significant difference between the chamomile and placebo groups in terms of the severity of nausea and the frequency of vomiting immediately after recovery (time of zero), 1, 2, and 6 hours after surgery (P > 0.05). However, 4 hours post-surgery, the severity of nausea in the chamomile group was lower than in the placebo group, and a statistically significant difference was observed between the two groups (P = 0.03). No gastrointestinal side effects were reported.

**Conclusions:**

Based on the results of this study, chamomile can be used to reduce nausea and vomiting after middle ear surgery, given its availability and low cost.

## 1. Background

Postoperative nausea and vomiting (PONV) is a commonly observed complication within 24 hours after anesthesia, particularly prevalent in surgeries such as those involving the middle ear (1). Statistics indicate that PONV occurs in 20 - 30% of surgeries overall, with a notably higher incidence of 62 - 80% in middle ear surgeries (2). Middle ear surgery is associated with a high risk of PONV due to the stimulation of the vestibular labyrinth, which is innervated by the vestibular part of cranial nerve VIII (vestibulocochlear). This, in turn, activates the chemoreceptor trigger zone (CTZ) in the area postrema. Manipulation of the CTZ leads to the activation of the paracellular plexus formation, which is considered the emesis center and ultimately leads to vomiting. Additionally, parasympathetic nerve stimulation of the external ear, tympanic membrane, middle ear, and inner ear, which are supplied by cranial nerves V (trigeminal), VII (facial), VIII (vestibular), IX (glossopharyngeal), and X, can contribute to PONV (3). This event poses substantial risks to patients, including potential complications such as aspiration, dehydration, electrolyte imbalance, disruption of surgical incisions, hypertension, tachycardia, and prolonged length of stay in the Post-Anesthesia Care Unit (3, 4). In contemporary medical practice, several prophylactic methods are employed to reduce the incidence of PONV, including the use of 5HT3 antagonists, metoclopramide, and dexamethasone (5-8). Notably, of the 252 herbal medicines listed by the World Health Organization (WHO), 11% originate from plants, underscoring the substantial utilization of plant-derived compounds in medicine. It is estimated that around 25% of prescribed drugs worldwide are derived from plant sources. These products are often more cost-effective, widely accepted by communities, and more readily available (9).

*Matricaria chamomilla* (chamomile), for instance, contains compounds such as flavonoids, sesquiterpenes, coumarins, and polysaccharides. Its flowers are particularly rich in sugars, flavonoids, mucilages, phenyl carboxylic acids, amino acids, choline, and salts. Numerous studies have demonstrated its efficacy in offering antiemetic, anxiolytic, anticonvulsant, and antidiarrheal properties. It has also been found to be beneficial in addressing conditions such as motion sickness, indigestion, and even anorexia (10). Extracted aqueous chamomile extract exerts its effect by suppressing COX-2 gene expression, directly inhibiting the COX-2 enzyme, and reducing PGE2 levels in the RAW 264.7 cell line activated by lipopolysaccharide (at concentrations of 5 to 40 μg/mL). According to studies, the mechanism of chamomile’s effect is attributed to its inhibition of PGE2 production. Chamomile inhibits the production of PGE2 by suppressing COX-2 gene expression and directly inhibiting COX-2 enzyme activity. In fact, chamomile works with the same mechanism of action as non-steroidal anti-inflammatory drugs (11). 

## 2. Objectives

Given the frequent occurrence of PONV following middle ear surgeries, as highlighted in several studies (11-13), and the presence of conflicting findings regarding the impact of chamomile on nausea and vomiting, this study seeks to investigate the specific effect of chamomile on PONV in patients undergoing middle ear surgery.

## 3. Methods

This study was a triple-blind clinical trial registered on the Iran Registry of Clinical Trials under the code IRCT20210109049973N1, approved by the Ethics Committee of Iran University of Medical Sciences (IR.IUMS.REC.1400.378). The study was conducted on 110 eligible patients scheduled for middle ear surgeries (specifically tympanoplasty and mastoidectomy) at Hazrat-E Rasoul-E Akram and Amiralam hospitals in Tehran, Iran, from November to December 2023. Written informed consent was obtained from all participants prior to the intervention. Furthermore, this study complies with the Helsinki Declaration of 1973 (revised in 2013) regarding Ethical Principles for Medical Research Involving Human Subjects.

### 3.1. Patient Selection and Treatment

A total of 110 patients who were candidates for middle ear surgery at Rasul Akram Hospital (PBUH) were enrolled in the study based on the below formula and (Z_1-a/2_ = 1.96, Z_1-β_ = 0.84), according to the study by Borhan et al. (as cited by Rajabalizadeh et al.) (14). 


$$\frac{{\;\;\left(Z_{1-\frac{a}{2}}\;\;\sqrt{p_{1\;}q_{1}-p_{2\;}q_{2\;}}\;\;+Z_{1-\beta }\;\;\;\sqrt{2pq\;}\;\;\;\;\right)}^{2}}{d^{2}}$$

The inclusion criteria consisted of individuals aged 18 - 65, classified as ASA I and II, who had not taken antiemetic medication 24 hours prior to surgery, exhibited a platelet count greater than 100,000 per microliter, had a body weight under 90 kg, had no hepatic disease, were not pregnant, had no renal diseases such as renal stones or renal failure, were free from gastrointestinal diseases such as gastric or duodenal ulcers, had no history of hepatitis, motion sickness, cancer, or hypertension, were in a non-per-oral (NPO) state, had no history of seizures, were non-smokers, had no allergy to chamomile, and did not use anticoagulant drugs or central nervous system (CNS) depressants. The exclusion criteria included the need for antiemetic medication during the intervention and patients who were unwilling to participate. Patients were randomly assigned to two groups using a coin-tossing method. The intervention group was labeled with the letter A, and the control group with the letter B, to prevent bias in data collection and result recording, thereby ensuring the generalizability of the findings.

According to the effective dose identified in published studies, including the study by Pakniat et al. in 2018, chamomile has been used and exhibited satisfactory results (15). In the pharmacology laboratory of Zardband Pharmaceutical Company, chamomile medication with the code 171 was prepared as an extract using a method approved by the Iranian Food and Drug Organization. To extract the chamomile, a pharmacognosy specialist used 20 g of chamomile flower powder in 200 mL of 70% ethyl alcohol. After 48 hours of preservation, the solution was filtered and then placed in a rotary machine at a temperature of 75°C for extraction.

For blinding, in accordance with the three-way blind approach of the study, capsules with the same shape were placed into two envelopes by the pharmacist. The person who performed the sampling, the patient who received the drug, and the statistical consultant who conducted the data analysis were all unaware of the allocation of the samples to the intervention and control groups. The intervention group was labeled with the letter A, and the control group was labeled with the letter B to prevent bias in data collection and result recording, ensuring the generalizability of the findings.

**Figure 1.**
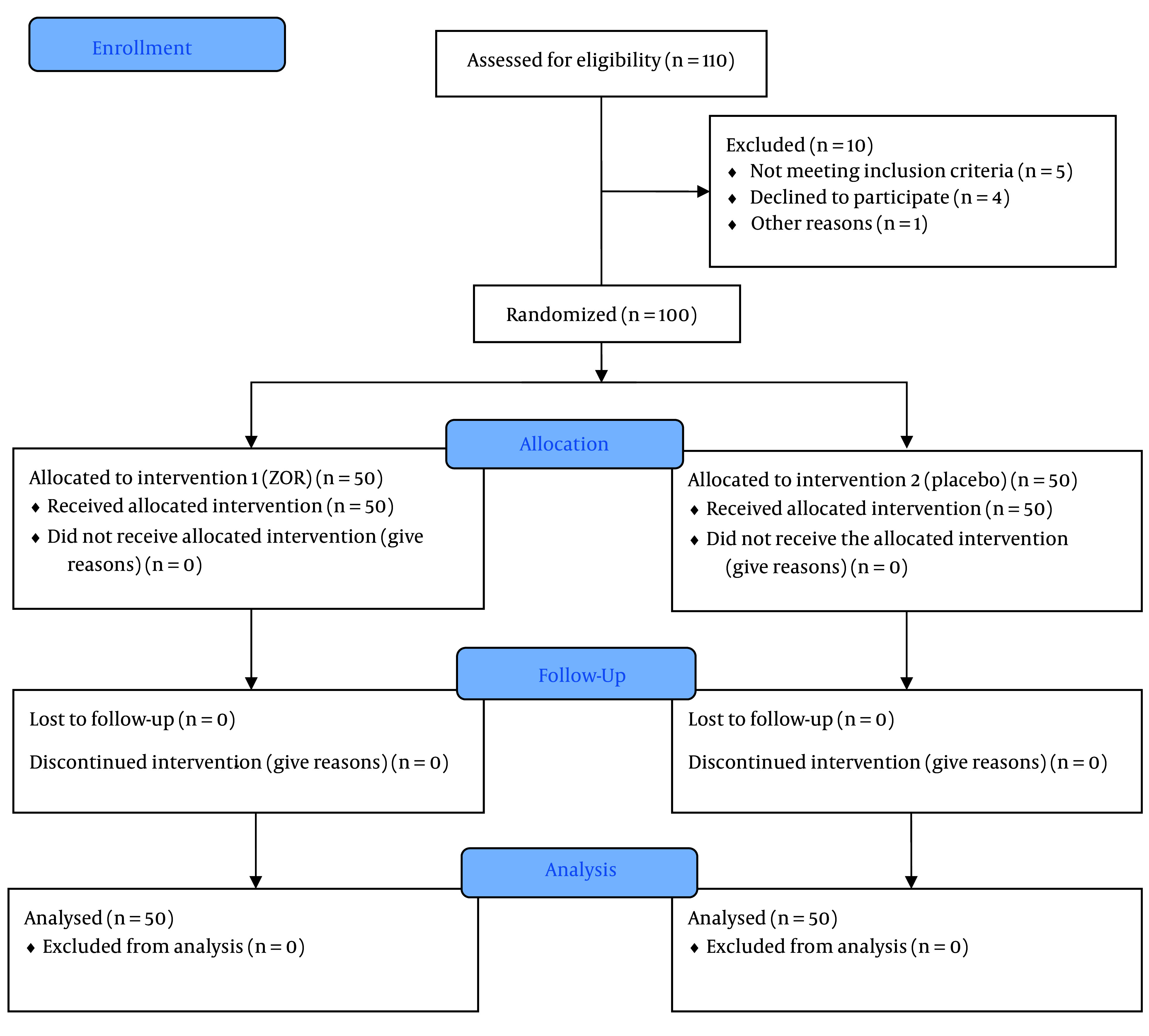
The CONSORT diagram

In line with the triple-blind approach, capsules prepared in a similar form by the pharmacist were placed in two identical packets. In the intervention group (group A), 50 patients received chamomile capsules, a product of Zardband Pharmaceuticals, with the registration license for the supplement numbered 70069006810023 and factory standard 00001/81/65, one hour before surgery, at a dose of 500 mg. In the control group (group B), 50 patients received a placebo (cornstarch) one hour before surgery. Both groups took the capsules with 30 mL of distilled water. Figure 1 illustrates the CONSORT diagram for study participants, providing a clear overview of the number of participants at each stage and the reasons for any exclusions or losses to follow-up.

In this study, the approximate duration of anesthesia, the anesthetic agents used, and the anesthesia technique employed were the same for all participants, and drugs such as morphine were avoided due to the possibility of inducing nausea and vomiting. To induce anesthesia, the following drugs were used: Midazolam 2 mg, fentanyl 2 µg/kg, thiopental 5 mg/kg, and atracurium 0.5 mg/kg. Propofol infusion was used to maintain anesthesia. If the patients experienced more than two episodes of vomiting, ondansetron 2 mg IV PRN was administered.

### 3.2. Primary and Secondary Aims 

The primary aim of this study was to investigate the effectiveness of chamomile in reducing the incidence and severity of nausea and vomiting after middle ear surgery. The secondary aims included determining the relationship between the severity of nausea and vomiting after middle ear surgery and the administration of chamomile capsules, with respect to the background and demographic variables of the patients.

### 3.3. Data Gathering and Statistical Analysis 

Immediately after the completion of the surgical operation and recovery from anesthesia, and then at 1, 2, 4, and 6 hours post-recovery (a total of five times), patients completed the valid and reliable Rhodes Index Questionnaire based on their symptoms. The Rhodes Index is a measure for nausea and vomiting, consisting of eight questions, and scored using a 5-point Likert Scale (ranging from 0 to 4). Three of these questions pertain to the frequency, severity, and duration of nausea, which are scored on a scale from 0 to 12. Two questions focus on retching, including its frequency and severity, with a range of 0 to 8. The total score of this scale ranges from 0 to 32. A score of 3 - 8 indicates mild nausea and vomiting, 9 - 16 indicates moderate, 17 - 24 indicates severe, and 25 - 32 is considered very severe (16) (Table 1). 

**Table 1.** The Rhodes Index

Scores	0	1	2	3	4
**In the last 12 hours, I threw up ------- times**	I did not throw up	1 - 2	3 - 4	5 - 6	≥ 7
**In the last 12 hours from retching and dry heaves I have felt ----- distress**	No	Mild	Moderate	Great	Sever
**In the last 12 hours from vomiting or throwing up, I have felt ----- distress**	No	Mild	Moderate	Great	Sever
**In the last 12 hours, I have felt nauseated or sick in my stomach ----- times** (h)**.**	Not at all	≥ 1	2 - 3	4 - 6	≥ 6
**In the last 12 hours, from nausea/sickness to my stomach, I have felt ----- distress.**	No	Mild	Moderate	Sever	Great
**In the last 12 hours, each time I threw up i produced a ----- amount**	I did not throw up	Small	Moderate	Large	Very large
**In the last 12 hours, I have felt nauseated or sick in my stomach ----- times.**	No	1 - 2	3 - 4	5 - 6	≥ 7
**In the last 12 hours, I have had periods of retching/dry heaves without bringing up ----- times**	No	1 - 2	3 - 4	5 - 6	≥ 7

After data collection and completion of the questionnaires, the data were entered into SPSSv.21 software by a statistical consultant and analyzed using various statistical tests. For the descriptive section, measures such as the mean, standard deviation, and frequency were used. To analyze the data, the following statistical tests were performed: The *t*-test to compare age and Rhodes Index scores between the two groups, the Mann-Whitney U test to compare the severity of nausea between the two groups, and the chi-square test to compare the frequency of vomiting between the two groups.

## 4. Results 

In this study, 110 patients (60% male and 40% female) were included. Ten patients were excluded from the study due to failure to meet the inclusion criteria or other reasons. Biometric, demographic, and surgery data are presented in Table 2. 

**Table 2.** Individual Characteristics in Chamomile and Placebo Groups ^a^

Indices	Chamomile (N = 50)	Placebo (N = 48)	P-Value
**Age (y)**	36.14 ± 10.3	34.28 ± 13.3	0.07
**Gender**			0.08
Male	22	23	
Female	23	22	
**Weight (kg)**	70.68 ± 2.10	70.06 ± 3.02	0.55
**Surgery duration (min)**	31.8 ± 10.7	36.7 ± 10.6	0.06
**Systolic blood pressure (mmHg)**	113.9 ± 3.6	124.6 ± 14.9	0.23
**Diastolic blood pressure (mmHg)**	80.57 ± 17.84	76.93 ± 20.76	0.86
**Heart rate (per min)**	80 ± 6.71	74 ± 7.12	0.74
**Respiratory rate (per min)**	13.21 ± 10.7	14.93 ± 1.71	0.25
**Body temperature (°C)**	36.18 ± 3.59	36.84 ± 1.33	0.53
**Times anesthesia (h)**	1.86 ± 0.63	1.38 ± 0.23	0.52
**History of PONV**	1	0	0.08

^a^ Values are expressed as mean ± SD unless otherwise indicated.

**Figure 2.**
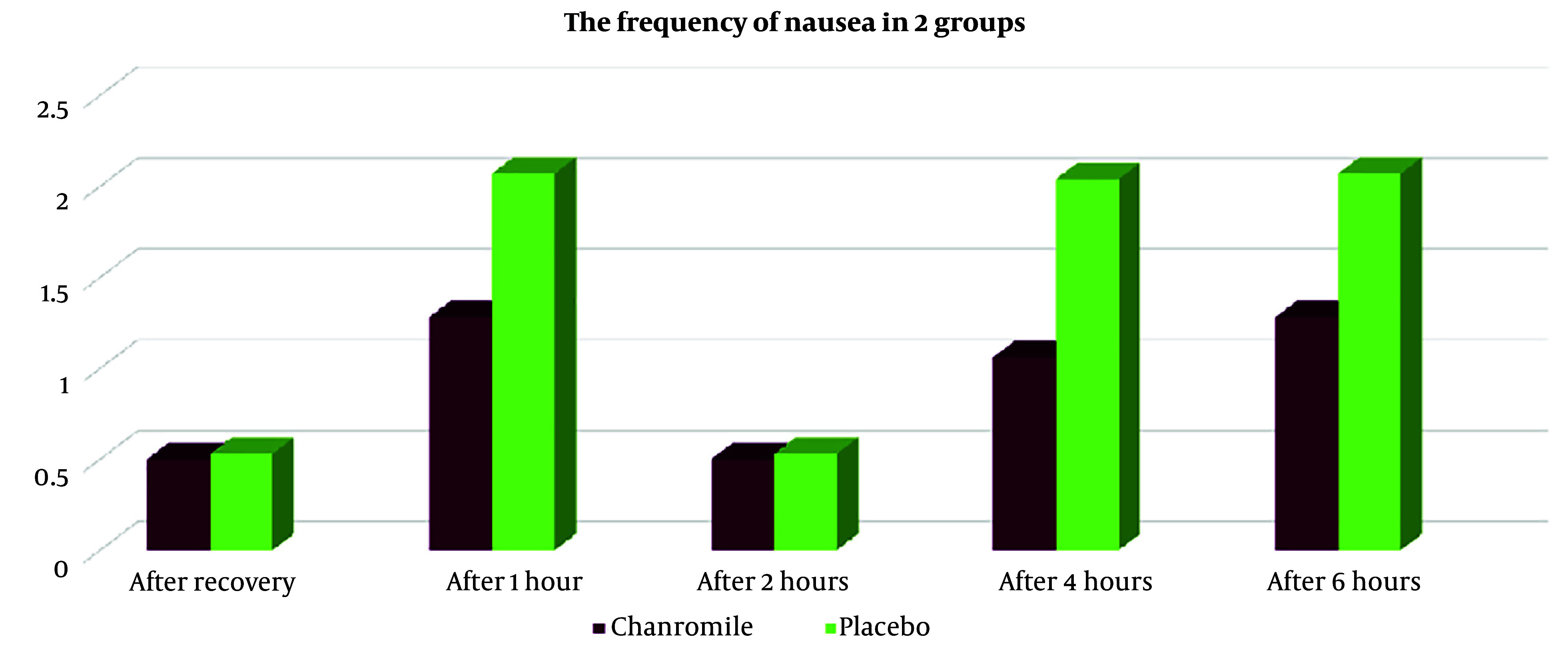
The frequency of nausea in the two groups

**Figure 3.**
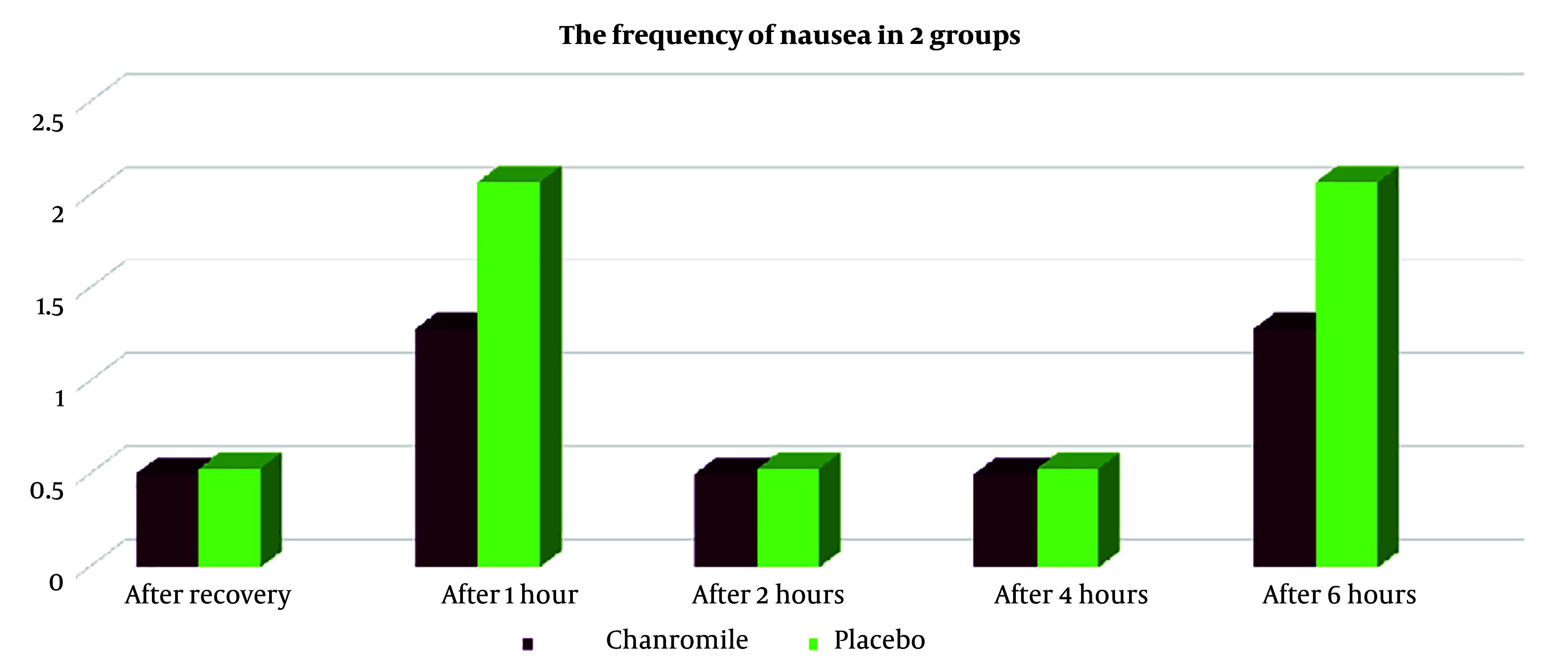
The frequency of vomiting in the two groups

Based on the results shown in Table 3, seven patients (14%) in group A reported nausea, compared to twenty patients (40%) in group B (P < 0.005). The Rhodes Index for nausea and vomiting, measured immediately after recovery, as well as at 1 hour, 2 hours, 4 hours, and 6 hours after recovery, was lower in group A than in group B. However, the frequency of nausea at 4 hours after recovery showed statistically significant differences between the two groups (Figure 2). Additionally, vomiting in group A was lower immediately after recovery (time of zero), at 1 hour, 2 hours, 4 hours, and 6 hours after recovery (Figure 3, Tables 3, and 4), although these differences were not statistically significant (P > 0.005).

**Table 3.** The Frequency of Nausea in the Two Groups

Nausea	Chamomile Group (N = 50)	Placebo Group (N = 50)	P-Value
**After recovery (time of zero)**			0.8
Mean ± SD	0.50 ± 1.42	0.53 ± 1.33	
No. (%)	0 (0)	2 (4)	
**After 1 hour**			0.1
Mean ± SD	1.28 ± 2.47	2.07 ± 2.79	
No. (%)	1 (5)	4 (8)	
**After 2 hours**			0.8
Mean ± SD	0.50 ± 1.40	0.53 ± 1.33	
No. (%)	0 (0)	2 (4)	
**After 4 hours**			0.03
Mean ± SD	1.06 ± 2.05	2.04 ± 2.79	
No. (%)	5 (10)	8 (16)	
**After 6 hours**			0.1
Mean ± SD	1.28 ± 2.47	2.07 ± 2.79	
No. (%)	1 (5)	4 (8)	

**Table 4.** The Frequency of Vomiting in the Two Groups

Nausea	Chamomile Group (N = 50)	Placebo Group (N = 50)	P-Value
**After recovery (time of zero)**			0.8
Mean ± SD	0.50 + 1.42	0.53 + 1.33	
No. (%)	0 (0)	2 (4)	
**After 1 hour**			0.1
Mean ± SD	1.28 + 2.47	2.07 + 2.79	
No. (%)	1(5)	4 (8)	
**After 2 hours**			0.8
Mean ± SD	0.50 + 1.40	0.53 + 1.33	
No. (%)	0 (0)	2 (4)	
**After 4 hours**			0.8
Mean ± SD	0.50 + 1.40	0.53 + 1.33	
No. (%)	0 (0)	2 (4)	
**After 6 hours**			0.1
Mean ± SD	1.28 + 2.47	2.07 + 2.79	
No. (%)	1 (5)	4 (8)	

As shown in Table 2, individual characteristics, including demographic data, vital signs (heart rate, systolic and diastolic blood pressure, respiratory rate), and the duration of anesthesia in the two groups, revealed no significant differences (P > 0.5).

## 5. Discussion 

Nausea and vomiting after surgery are among the most important side effects of anesthesia and surgery (8, 17). Although anti-nausea drugs are used preventively, the occurrence of side effects from these drugs remains a challenge, similar to other chemical medications (18). Some predictors of nausea and vomiting after surgery include age, gender, anxiety, prolonged surgery, use of sedatives, anesthesia, and more (19-21). Chamomile is a medicinal plant found in many Asian countries (22). The anti-nausea and anti-vomiting effects of this herbal medicine have been reported and confirmed in several studies (15, 23, 24). The present study aimed to investigate the effect of chamomile capsules on the incidence and severity of nausea and vomiting after middle ear surgery. 

Based on the results of this study, the overall incidence of nausea was similar in both groups; however, the severity and likelihood of nausea differed between the chamomile and placebo groups. In a study conducted by Pakniat et al. in Qazvin in 2016, the effects of chamomile, ginger, and vitamin B6 on the treatment of nausea and vomiting during pregnancy were compared. The results of this study indicated that all three treatments—chamomile, ginger, and vitamin B6—were effective in reducing nausea and vomiting during pregnancy, with medications administered over a 4-day period (15). This finding helps justify the effectiveness of chamomile in our study. In contrast, our study involved administering the oral capsule only once, prior to the surgery. It is possible that more frequent administration could lead to more pronounced effects of this herbal medicine.

In this regard, in 2014, Borhan et al. (as cited by Rajabalizadeh et al.) conducted a quasi-experimental study on the effect of chamomile plant extract on the severity of chemotherapy-related nausea and vomiting in 60 patients undergoing chemotherapy at the Social Security Hospital in Zahedan. In this study, chamomile extract was administered to the intervention group two hours before chemotherapy. The results concluded that chamomile extract reduced nausea caused by chemotherapy but was not effective in reducing vomiting (14). 

In a triple-blind clinical trial conducted by Modares et al. in 2018 in Gorgan, the effects of oral ginger and chamomile capsules on nausea and vomiting during pregnancy were compared. In this study, pregnant women took 500 mg of ginger or chamomile capsules twice a day for one week. The results showed that chamomile capsules were more effective than ginger in reducing nausea and vomiting during pregnancy (25). The dose used in this study is similar to the dose used in the present study. Therefore, it may be concluded that more time may be needed after administering the drug for better effectiveness of chamomile. According to the results of the current study, 4 hours after regaining consciousness, the severity of nausea and vomiting in the chamomile group significantly decreased.

In a study conducted by Sanaati et al. in 2013 - 2014, the effect of ginger and chamomile on nausea and vomiting in Iranian women with breast cancer undergoing chemotherapy was investigated. The patients were divided into three groups: Ginger, chamomile, and control. The ginger group received 500 mg capsules of powdered ginger root twice a day for 5 days before and after chemotherapy, in addition to a standard antiemetic regimen. The chamomile group received a similar regimen with chamomile extract capsules, while the control group received only the standard antiemetic regimen. Both ginger and chamomile were found to reduce the frequency of vomiting after chemotherapy, with ginger also showing a reduction in nausea. These findings are consistent with the results of the present study (24). 

In a cross-sectional study conducted by Alonso-Castro et al. in Mexico in 2016 - 2017, the effectiveness of chamomile for reducing nausea and vomiting during pregnancy was also reported (26). 

In general, there are very few studies on the effect of chamomile capsules on nausea and vomiting after surgery. Therefore, more extensive studies are needed to further investigate the effectiveness of this herbal medicine. In the future, it may be possible to suggest that chamomile be included as an adjunct or in the patient's medication regimen, even a few days before surgery, to reduce the incidence and severity of PONV. 

In conclusion, the findings of this study revealed that there was no significant difference in the severity of nausea and the frequency of vomiting between the chamomile and placebo groups immediately after awakening from anesthesia and at 1, 2, and 6 hours post-operation. However, at the 4-hour mark, the chamomile group exhibited lower nausea severity compared to the placebo group, indicating a significant difference. Furthermore, no gastrointestinal side effects were reported in either group. These results suggest that chamomile may be a safe and cost-effective option for reducing PONV following middle ear surgery. Further research is recommended to explore the potential benefits of chamomile in this context.

## Data Availability

The dataset presented in this study is available upon request from the corresponding author during submission or after publication. The data are not publicly available due to patient privacy concerns.

## Clinical Trial Registration Code:

This study was a triple-blind clinical trial registered on the Iran Registry of Clinical Trials under the code IRCT20210109049973N1.
